# Making a good model better - evaluation of the NSW Combined Family Cancer Clinic meetings

**DOI:** 10.1186/1897-4287-10-S2-A53

**Published:** 2012-04-12

**Authors:** Lynne Purser, Kathy Tucker, Kristine Barlow-Stewart, Kate Dunlop

**Affiliations:** 1 Centre for Genetics Education, NSW Health, Sydney, NSW, Australia; 2 Hereditary Cancer Centre, Prince of Wales Hospital, Randwick, NSW, Australia; 3 Northern Clinical School, Sydney Medical School, University of Sydney, NSW, Australia

## 

Ensuring best practice in the NSW/ACT Family Cancer Service has considerable challenges given the geographical dispersion of geneticists, oncologists and genetic counsellors and the small often isolated teams in which they operate. The combined monthly meetings of 25-30 genetic health professionals were first initiated by a senior geneticist in partnership with the Centre for Genetics Education in 1996 in order to share expertise, keep abreast of rapidly developing evidence, and provide a network of support. The 3 hour meetings have been well attended, with four meetings per year via video/ teleconference. A previous evaluation was conducted in 2004. New IT technologies are providing flexibility in conferencing and an increased demand on genetic services means time away from the workplace for meetings may be less cost effective. Consequently a recent evaluation was undertaken to identify the perceived outcomes of the meeting and inform future planning and funding.

A questionnaire requesting anonymous participant views on the current format, impact of the meetings on clinical practice, preferred mode of meeting and ideas for future development was distributed. 22/25 (88%) surveys were completed (though 2 were invalid). Overall, everyone was satisfied with the meeting experience and format with 90% very to extremely satisfied. 85% of participants agreed or agreed strongly that the meetings impacted positively on their clinical practice. Perceived outcomes of the meeting included: expert opinion gained on complex cases; successful avenue for updating knowledge of current literature; engagement with relevant literature through journal presentations; enhancement of clinical practice through case presentation and discussion and enhancement of patient outcomes through networking. Despite the opportunities available through IT, participants reported the inconsistency in delivery of videoconferences and the preference for face-to-face meetings. Participant comments included: *‘This is a brilliant meeting that is well worth attending each month.’; ‘Excellent opportunity to review difficult cases & go back to a patient/family with confidence.’; ‘I think these meetings help cohesion in cancer genetics - uniformity’.* The perceived impact on clinical practice by participants and the high level of satisfaction is a strong endorsement for the continuation of the FCC meetings.

